# A Single-Center Analysis of Sex Differences in Patients With Chronic Subdural Hematoma in China

**DOI:** 10.3389/fneur.2022.888526

**Published:** 2022-05-17

**Authors:** Yunwei Ou, Wenhua Fan, Xiaofan Yu, Liang Wu, Weiming Liu

**Affiliations:** ^1^Department of Neurosurgery, Beijing Tiantan Hospital, Capital Medical University, Beijing, China; ^2^Hefei Comprehensive National Science Center, The Institute of Artificial Intelligence, Hefei, China; ^3^China National Clinical Research Center for Neurological Diseases, Beijing, China; ^4^Beijing Advanced Innovation Center for Big Data-Based Precision Medicine, Beihang University, Beijing, China; ^5^Department of Molecular Neuropathology, Beijing Neurosurgical Institute, Capital Medical University, Beijing, China; ^6^Neurological Center, People's Hospital of Ningxia Hui Autonomous Region, Yinchuan, China

**Keywords:** clinical characteristic, chronic subdural hematoma, difference, gender, surgery, age stratification

## Abstract

**Background:**

Given the men's predominance in the prevalence of chronic subdural hematoma (CSDH), we investigated the relationship between sex differences and clinical features of CSDH.

**Methods:**

We retrieved a large collection of clinical factors from CSDH patients between August 2011 and May 2019, and analyzed the differences and similarities in the clinical data and outcomes between men and women.

**Results:**

In total 1,307 CSDH patients were enrolled in this study. When we did not account for age, a greater proportion of women relative to men manifested diabetes (*p* = 0.001) and cardiac disease (*p* = 0.035) prior to the onset of CSDH. Regarding recovery outcome and recurrence rate, we observed no significant differences between men and women. The sole difference between women and men after surgery was that women experienced more complications than men (*p* = 0.044), and both length of hospital stay (*p* < 0.001, B = 0.159, Exp [B] = 1.172, 95% CI = 1.078–1.274) and the presence of cardiac disease (*p* = 0.002, B = 2.063, Exp [B] = 7.867, 95% CI = 2.167–28.550) were identified as independent risk factors. After accounting for age, women with CSDH exhibited more frequent disorders of consciousness at admission than men in group of ≤ 40-year-old patients (*p* = 0.018), while proportion of women with diabetes was higher than that of men in 41–79 year-old group (*p* < 0.001). However, women after surgery experienced more complications (*p* = 0.047), longer length of hospital stays (*p* = 0.005), and higher mortality at discharge (*p* = 0.035) than men in middle-aged group. Finally, length of hospital stay (*p* < 0.001, B = 0.186, Exp [B] = 1.205, 95% CI = 1.091–1.331) and cardiac disease (*p* = 0.017, B = 2.040, Exp [B] = 7.693, 95% CI = 1.430–41.372) impacted occurrence of complications in women 41–79-year-old, while duration of drainage catheter use (*p* < 0.001, B = 1.132, beta = 0.280) and complications (*p* < 0.001, B = 5.615, beta = 0.366) were identified as independent risk factors for length of hospital stay in the same group of women.

**Conclusions:**

Although sex differences did not constitute a crucial factor in all the CSDH patients, we still need to pay closer attention to disparities between men and women with respect to complications, length of hospital stay, and mortality at discharge in the various age groups (particularly with respect to 41–79 year-old women patients), to provide satisfactory management and treatment of CSDH patients.

## Introduction

Chronic subdural hematoma (CSDH) is a prevalent disease seen in neurosurgical practices among older adults, and its annual incidence is rising commensurately as our population ages (1–5). According to recent studies, men-women differences have been reported to play an important role in either the characteristics of intracerebral hemorrhage or in outcomes following the neurosurgical intervention (6–8). Concerning sex-related disparities in CSDH, several large surveys from various countries have reached a consensus showing a male predominance in the prevalence of CSDH, with the men: women ratio ranging from 2:1 to 5:1 (9–14). Hotta et al. reported that in addition to women presenting with a greater incidence of disorders of consciousness at admission, the female sex also predicted a worse prognostic outcome and a significantly higher mortality rate at discharge (15). In contrast, Wang et al. uncovered no statistical difference between men and women patients regarding diminished consciousness resulting from CSDH, and they determined that men exhibited significantly increased mortality due to CSDH (13). Furthermore, several studies revealed that men suffered a higher recurrence rate than women after surgical intervention (16, 17). However, a majority of the literature (including our previous study) showed that sex differences were not associated with the recurrence rate (18–25).

We note from the available evidence that controversy remains regarding the relationship between sex differences and several clinical characteristics of CSDH as well as postsurgical outcomes—including disorders of consciousness observed upon admission, recurrence rate, and prognosis. Given that few studies have focused on this topic, we herein wished to clarify whether a sex difference existed for the clinical characteristics and therapeutic outcomes associated with patients manifesting CSDH.

## Materials and Methods

### Collection of Patient Data

In this single-center retrospective analysis, 1,307 patients with symptomatic CSDH were admitted to the Department of Neurosurgery at Beijing Tiantan Hospital, China between August 2011 and May 2019, and were followed up for 6 months were eligible for inclusion. Patients who underwent craniotomy or did not possess complete medical records were excluded. All the patients were diagnosed by pre-operative computed tomography (CT) or magnetic resonance imaging (MRI), followed by treatment with burr-hole craniotomy and a closed drainage system. According to the preoperative symptoms and signs, Bender-grade scoring was employed to analyze patient preoperative status (26). The demographic characteristics from each patient were collected by reviewing the clinical records retrospectively; these included age, sex, history of head trauma, personal or past history, use of anticoagulant or antiplatelet medications, clinical symptoms, and comorbidities such as hypertension, diabetes mellitus, brain infarction, and cardiovascular disease. We then used follow-up CT characteristics of pre-operative and post-operative conditions at 6 months to calculate hematoma volumes by using the coniglobus formula. Additionally, the modified Rankin scale (MRS) score at 6 months was exploited to analyze the outcome after surgery. We ultimately analyzed the postoperative CSDH recurrence rate by collecting the reoperation rate.

This protocol was implemented with the approval of our Institutional Research Ethics Committee (no. KY 2020-094-02) and written informed consent was obtained prior to surgery from the patients themselves or from their closest living relatives.

### Statistical Analysis

We performed statistical analyses with SPSS software (version 17.0.0; SPSS Inc., Chicago, IL). Continuous variables were described as means (± standard deviations) and categorical variables were presented as the numbers of patients (percentages). The relationships between clinical characteristics and prognostic outcomes in men and women were assessed using the Student's *t*-test and Chi-squared test. We also executed univariate and multivariate logistic regression analyses to analyze the factors related to sex differences. Only the significant factors in the univariate analysis were used for multivariate analysis. *p* < 0.05 was considered to be significant.

## Results

### Baseline Clinical Characteristics of CSDH Patients

In this single-center, large-sample, analytic cohort study, we included and treated a total of 1,307 consecutive patients with CSDH between August 2011 and May 2019. 1,080 men (82.6%) and 227 women (17.4%). This sex ratio was consistent with that reported in previous studies and highlighted the first characteristic of male predominance in CSDH. The patient's mean age was 63.1 ± 16.9 years, and 61.5% (805/1,307) of patients had experienced head trauma. With respect to patient history prior to CSDH onset, we noted that 30.5% (399/1,307) of patients were diagnosed with coagulatory dysfunction, while only 11.0% (144/1,307) of patients received antithrombotic (antiplatelet or anticoagulant) medication—indicating that there were some arcane factors responsible for the preoperational coagulation abnormality. Additionally, there were 26.7 and 19.0% of patients showed smoking and drinking history, respectively. Hypertension was the most common comorbidity, followed by diabetes, brain infarction, and cardiac disease. A vast majority of patients (94.7%) manifested CSDH of primary onset, with an interval from onset to the admission of 24.1 ± 30.3 days—exhibiting the chronic onset characteristic of CSDH. We observed unilateral hematomas in 963 patients (left-and-right aspects, 41.4 and 32.3%), while 26.3% of patients exhibited bilateral hematomas. Employing the coniglobus formula, we calculated a preoperative hematoma volume of 99.5 ± 30.0 ml. All the patients showed the common presenting symptoms of headache (58.0%) and limb weakness (54.9%), followed by dizziness (26.9%), dysphasia (9.3%), and disorders of consciousness (4.6%).

When we evaluated sex differences in the clinical characteristics of patients with CSDH, we noted a remarkable predominance of men (1,080 men vs. 227 women; *p* < 0.001) (Table 1). The sex ratios with respect to smoking and drinking history were also significantly skewed toward men (all *p* < 0.001), consistent with a previous report (15), while the women patients exhibited greater incidence rates of diabetes and cardiac disease (*p* = 0.001 and *p* = 0.035, respectively). Notably, in contradistinction to the results of Hotta et al., no difference was observed in patient disorders of consciousness at admission between men and women (*p* = 0.11). Although the symptom of headache was more common in men than in women (*p* = 0.002), there were no significant differences in any other preoperative clinical characteristic—including Bender grades, head trauma experience, or preoperative hematoma volume or location. We also analyzed the relationship between clinical characteristics and sex in CSDH patients of different ages: a ≤ 40 year-old group (*n* = 126; women vs. men = 15 vs. 111), a group 41–79 years of age (*n* = 1,002; women vs. men = 166 vs. 836), and a ≥ 80 year-old group (*n* = 179; women vs. men = 46 vs. 133)—and demonstrated male predominance in all of the age groups (*p* < 0.001). As shown in Table 2, the proportions of patients with smoking or drinking history were significantly higher in men than in women aged 41–79 years (*p* < 0.001) and ≥ 80 years (*p* < 0.001, *p* = 0.010), while the ≤ 40-year-old group was not different. Intriguingly, we uncovered a greater incidence of disorders of consciousness at admission in women than in men aged ≤ 40 years (*p* = 0.018), with no significant differences in the other age groups. We also found that the proportion of women patients with diabetes was elevated relative to that of men between 41 and 79 years of age (*p* < 0.001).

**Table 1:** The clinical characteristics and relationship between clinical characteristics and groups of gender in patients with chronic subdural hematoma.

**Characteristics analyzed**		**Groups of gender (** ***n*** **)**			***p***
	**Patients *n* (%)**	**Women (227)**	**Men (1,080)**		
Personal/past history					
Smoking	349 (26.7)	11 (4.8)	338 (31.3)	<0.001	
Drinking	249 (19.0)	9 (4.0)	240 (22.2)	<0.001	
Hypertension	467 (35.7)	89 (39.2)	378 (35.0)	0.229	
Diabetes	257 (19.6)	63 (27.8)	194 (18.0)	0.001	
Cardiac diseases	49 (3.7)	14 (6.2)	35 (3.2)	0.035	
Brain infarction	143 (10.9)	29 (12.8)	114 (10.6)	0.330	
History of antithrombotic	144 (11.0)	23 (10.1)	121 (11.2)	0.639	
AC/V-P shunt n (%)	115 (8.8)	19 (8.4)	96 (8.9)	0.802	
Symptoms					
Headache n (%)	758 (58.0)	111 (48.9)	647 (59.9)	0.002	
Dizziness n (%)	351 (26.9)	65 (28.6)	286 (26.5)	0.506	
Limb weakness n (%)	717 (54.9)	130 (57.3)	587 (54.4)	0.422	
Dysphasia n (%)	122 (9.3)	28 (12.3)	94 (8.7)	0.087	
Disturbance of consciousness n (%)	60 (4.6)	15 (6.6)	45 (4.2)	0.110	
Bender grades					0.232
0	66 (5.0)	11 (4.8)	55 (5.1)		
I	556 (42.5)	89 (39.2)	467 (43.2)		
II	629 (48.1)	112 (49.3)	517 (47.9)		
III	56 (4.3)	15 (6.6)	41 (3.8)		
Head trauma event n (%)	805 (61.5)	83 (36.6)	419 (38.8)	0.530	
BMI	23.7 ± 4.6	22.9 ± 3.9		26 ± 5.8	0.349
Unilateral/bilateral hematoma					0.965
Left	541 (41.4)	93 (41.0)	448 (41.5)		
Right	422 (32.3)	75 (33.0)	347 (32.1)		
Bilateral	344 (26.3)	59 (26.0)	285 (26.4)		
Preoperative volume (ML)	99.5 ± 30.0	98.5 ± 29.7	99.7 ± 30.0	0.606	

**Table 2:** Relationship between clinical characteristics and groups of gender in different age patients with chronic subdural hematoma.

**Characteristics analyzed**	**≤40 years**			**41–79 years**			**≥80 years**		
	**Women (15)**	**Men (111)**	***p***	**Women (166)**	**Men (836)**	***p***	**Women (46)**	**Men (133)**	***p***
Personal/past history									
Smoking	1 (6.7)	23 (20.7)	0.299	9 (5.4)	277 (33.1)	<0.001	1 (2.2)	38 (28.6)	<0.001
Drinking	2 (13.3)	16 (14.4)	1.000	6 (3.6)	201 (24.0)	<0.001	1 (2.2)	23 (17.3)	0.010
Hypertension	2 (13.3)	4 (3.6)	0.150	68 (41.0)	316 (37.8)	0.444	19 (41.3)	58 (43.6)	0.786
Diabetes	0 (0.0)	1 (0.9)	1.000	55 (33.1)	151 (18.1)	<0.001	8 (17.4)	42 (31.6)	0.065
Cardiac diseases	0 (0.0)	0 (0.0)	-	8 (4.8)	28 (3.3)	0.353	6 (13.0)	7 (5.3)	0.100
Brain infarction	0 (0.0)	0 (0.0)	-	23 (13.9)	90 (10.8)	0.250	6 (13.0)	24 (18.0)	0.434
History of antithrombotic	0 (0.0)	0 (0.0)	-	16 (9.6)	90 (10.8)	0.519	3 (6.5)	17 (12.8)	0.553
AC/V-P shunt n (%)	8 (53.3)	65 (58.6)	0.700	9 (5.4)	28 (3.3)	0.196	2 (4.3)	3 (2.3)	0.604
Symptoms									
Headache n (%)	11 (73.3)	92 (82.9)	0.474	91 (54.8)	512 (61.2)	0.122	9 (19.6)	43 (32.3)	0.100
Dizziness n (%)	5 (33.3)	26 (23.4)	0.522	54 (32.5)	230 (27.5)	0.190	6 (13.0)	30 (22.6)	0.165
Limb weakness n (%)	2 (13.3)	16 (14.4)	1.000	92 (55.4)	462 (55.3)	0.970	36 (78.3)	109 (82.0)	0.582
Dysphasia n (%)	0 (0.0)	1 (0.9)	1.000	21 (12.7)	77 (9.2)	0.173	7 (15.2)	16 (12.0)	0.578
Disturbance of consciousness n (%)	4 (26.7)	6 (5.4)	0.018	7 (4.2)	31 (3.7)	0.754	4 (8.7)	8 (6.0)	0.531
Bender grades			0.237			0.408			0.788
0	2 (13.3)	10 (9.0)		7 (4.2)	40 (4.8)		2 (4.3)	5 (3.8)	
I	8 (53.3)	82 (73.9)		70 (42.2)	362 (43.3)		11 (23.9)	23 (17.3)	
II	2 (13.3)	12 (10.8)		80 (48.2)	410 (49.0)		30 (65.2)	95 (71.4)	
III	3 (20.0)	7 (6.3)		9 (5.4)	24 (2.9)		2 (6.5)	10 (7.5)	
Head trauma event n (%)	7 (46.7)	57 (51.4)	0.733	107 (64.5)	516 (61.7)	0.507	30 (65.2)	88 (66.2)	0.907
Unilateral/bilateral hematoma			0.520			0.893			0.765
Left	7 (46.7)	44 (39.6)		68 (41.0)	358 (42.8)		18 (39.1)	46 (34.6)	
Right	6 (40.0)	37 (33.3)		54 (32.5)	268 (32.1)		15 (32.6)	42 (31.6)	
Bilateral	2 (13.3)	30 (27.0)		44 (26.5)	210 (25.1)		13 (28.3)	45 (33.8)	
Preoperative volume (ML)	102.5 ± 29.6	92.1 ± 30.7	0.216	95.9 ± 30.7	98.8 ± 29.5	0.249	106.6 ± 24.9	111.2 ± 29.8	0.354

### Clinical Characteristics of CSDH Patients After Surgery

The patients subsequently underwent burr-hole craniotomy. In total 81.0% (1,059/1,307) were subjected to a unilateral operation (left: right=45.0%:36.0%); the volume of the hematoma cavity was reduced by an average of 57.5 ± 21.8 ml and the mean postoperative volume was 38.7 ±23.2 ml. During the therapeutic course, urokinase was injected into the hematoma cavity in 53.9% of patients after the operation, and the duration time for drainage catheter use was 3.4 ± 1.9 days. Further analysis indicated that the mean hospital stay was 7.7 ± 4.2 days, with only 6.7% (87/1,307) of patients experiencing postoperative complications. In addition, recurrence that required reoperation was 1.8% (24/1,307), and 0.6% (8/1,307) of patients died before discharge. We obtained follow-up information by telephone. Finally, after we evaluated patients with the MRS, we noted that a total of 1,061 (81.2%) patients achieved a favorable outcome (MRS score, 0), 78 patients had an acceptable outcome (MRS score, 1), and only 2.5% (32/1,307) of the patients presented a poor outcome (MRS score, 4–6). When we further analyzed the relationship between clinical outcomes and sex, as shown in Table 3, we determined that outcomes for the 1,307 patients as evaluated by MRS score (i.e., with respect to a favorable recovery outcome [MRS 0–3] or a poor outcome [MRS 4–6]) were not different between men and women, which did not support the concept that women manifested fewer instances of favorable recovery (15). The sole difference between women and men after surgery was that the women experienced more frequent complications than men (*p* = 0.044); the other characteristics after surgery were not different between men and women (Table 3).

**Table 3:** The clinical characteristics and relationship between clinical characteristics and groups of gender in patients with chronic subdural hematoma after surgery.

**Characteristics analyzed**	**Groups of gender (** ***n*** **)**			***p***
	**Patients *n* (%)**	**Women (227)**	**Men (1,080)**	
Reduction of hematoma cavity (%)	57.5 ± 21.8	57.8± 22.1	0.170	
Symptom disappeared or alleviated (day)	2.6 ± 2.1	2.7 ± 2.3	2.6 ± 2.0	0.510
Duration of drainage catheter (day)	3.4 ± 1.9	3.3 ± 1.4	3.5 ± 2.0	0.406
Length of hospital stay (days)	7.7 ± 4.2	8.1 ± 4.5	7.6 ± 4.1	0.081
Use of urokinase	705 (53.9)	113 (49.8)	592 (54.8)	0.167
Frequency of urokinase used	1.6 ± 0.9	1.6 ± 0.8	1.6 ± 0.9	0.941
Postoperative volume (mL)	38.7 ±23.2	36.1 ± 30.5	39.1± 28.1	
Recurrence requiring reoperation	24 (1.8)	6 (2.7)	18 (1.7)	0.317
Unilateral/bilateral operation				0.511
Left	589 (45.1)	101 (44.5)	488 (45.2)	
Right	470 (36.0)	88 (38.8)	382 (35.4)	
Bilateral	248 (19.0)	38 (16.7)	210 (19.4)	
Complications	87 (6.7)	22 (9.7)	65 (6.0)	0.044
Death (hospital stay)	8 (0.6)	3 (1.3)	5 (0.5)	0.146
Death (in 6 month)	18 (1.4)	6 (2.6)	12 (1.1)	0.107
Outcome (MRS)				0.507
0–3	1,266 (96.9)	217 (95.6)	1,049 (97.1)	
4–6	32 (2.4)	8 (3.5)	24 (2.2)	

We also identified the length of hospital stay (*p* < 0.001, B = 0.159, Exp [B] = 1.172, 95% CI = 1.078–1.274) and incidence of cardiac disease (*p* = 0.002, B = 2.063, Exp [B] = 7.867, 95% CI = 2.167–28.550) as independent risk factors for the onset of complications in women after surgery (Table 4). When we additionally assessed the relationships between clinical characteristics and sex within the various age groups of patients with CSDH after surgery, we observed more complications after surgery in women than in men patients aged 41–79 years (*p* = 0.005), while men and women in other age groups did not differ with respect to complications (Table 5). Interestingly, we also discovered that the women with CSDH experienced longer hospital stays (*p* = 0.047) and higher mortality at discharge than the men (women vs. men = 1.8 vs. 0.2, p=0.035) in the group comprised of patients 41–79 years of age.

**Table 4:** Logistic regression analyses of characteristics related to complications in women patients with chronic subdural hematoma.

**Characteristics analyzed**	**Women**				
	***p***	**B**	**Exp (B)**	**95% (CI)**	***p***
Age	0.370				
Smoking	1.000				
Drinking	0.605				
Hypertension	0.455				
Brain infarction	1.000				
History of antithrombotic	0.253				
Head trauma event	0.627				
Length of symptoms disappeared or alleviated	0.559				
BMI	0.721				
Primary onset	0.365				
Duration of drainage catheter	0.601				
Length of hospital stay	0.008	0.159	1.172	1.078–1.274	<0.001
Interval from onset to admission	0.745				
Reduction of hematoma cavity	0.800				
Diabetes	0.958				
Use of urokinase	0.983				
Frequency of urokinase used	0.762				
Unilateral/bilateral hematoma	0.543				
Unilateral/bilateral operation	0.863				
AC/VP shunt	1.000				
Headache	0.577				
Dizziness	0.882				
Limb weakness	0.276				
Dysphasia	0.490				
Disturbance of consciousness	1.000				
Cardiac diseases	0.001	2.063	7.867	2.167–28.550	0.002
Preoperative volume	0.090				
Postoperative volume	0.176				
Preoperative dysfunction of coagulation	0.287				

**Table 5:** Relationship between clinical characteristics and groups of gender in different age patients with chronic subdural hematoma after surgery.

**Characteristics analyzed**	**≤40 years**			**41–79 years**			**≥80 years**		
	**Women (15)**	**Men (111)**	***p***	**Women (166)**	**Men (836)**	***p***	**Women (46)**	**Men (133)**	***p***
Reduction of hematoma cavity (%)	53.6 ± 27.8	61.6 ± 23.6	0.229	55.6 ± 19.9	57.5 ± 22.0	0.293	57.3 ± 17.8	56.8 ± 21.4	0.883
Symptom disappeared or alleviated (day)	2.7 ± 3.0	2.4 ± 1.9	0.690	2.7 ± 2.2	2.6 ± 2.0	0.469	2.7 ± 2.3	2.9 ± 2.3	0.656
Duration of drainage catheter (day)	3.6 ± 2.4	3.4 ± 3.1	0.843	3.2 ± 1.2	3.4 ± 1.9	0.054	3.7 ± 1.6	3.6 ± 1.4	0.728
Length of hospital stay (days)	5.5 ± 2.0	7.5 ± 4.1	0.670	8.4 ± 4.8	7.6 ± 4.2	0.047	8.2 ± 3.8	7.7 ± 3.5	0.387
Use of urokinase	6 (40.0)	40 (36.0)	0.765	86 (51.8)	464 (55.5)	0.382	21 (45.7)	88 (66.2)	0.014
Frequency of urokinase used	1.3 ± 0.5	1.6 ± 0.8	0.862	1.6 ± 0.8	1.6 ± 0.9	0.486	1.8 ± 1.1	1.8 ± 1.1	0.092
Recurrence requiring reoperation	0 (0.0)	1 (0.9)	0.881	6 (3.6)	15 (1.8)	0.291	0 (0.0)	2 (1.5)	0.551
Complications	0 (0.0)	10 (9.0)	0.268	18 (10.8)	43 (5.1)	0.005	4 (8.7)	12 (9.0)	0.607
Death (hospital stay)	0 (0.0)	0 (0.0)	-	3 (1.8)	2 (0.2)	0.035	0 (0.0)	3 (2.3)	0.408
Death (in six month)	0 (0.0)	0 (0.0)	-	4 (2.4)	7 (0.8)	0.093	2 (4.3)	5 (3.8)	0.576
Outcome (MRS)			0.881			0.596			0.498
0–3	15 (100.0)	110 (99.1)		159 (95.8)	813 (97.2)		43 (93.5)	126 (94.7)	
4–6	0 (0.0)	1 (0.9)		5 (3.0)	16 (1.9)		3 (6.5)	7 (5.3)	

Based upon the aforementioned results, we further evaluated the predictors of complications in women patients with CSDH 41–79 years of age using logistic regression analysis. We calculated the clinical characteristics with a *p* < 0.05 in the univariate analysis, included the resultant variables in the multivariate analysis, and ascertained that the length of hospital stay (*p* < 0.001, B = 0.186, Exp [B] = 1.205, 95% CI = 1.091–1.331) and incidence of cardiac disease (*p* = 0.017, B = 2.040, Exp [B] = 7.693, 95% CI = 1.430–41.372) were independent risk factors and predictors of complication occurrence after surgery in the women aged 41–79 years (Table 6). We then analyzed the predictors of length of hospital stay in women with CSDH in the 41–79-year group by multiple linear regression analysis and demonstrated that the duration of drainage catheter use *(p* < 0.001, B = 1.132, beta = 0.280) and the incidence of complications (*p* < 0.001, B = 5.615, beta = 0.366) were independent risk factors and predictors of the length of hospital stay in the women patients in the middle-aged group (Table 7).

**Table 6:** Logistic regression analyses of characteristics related to complications in 41-79 years old women patients with chronic subdural hematoma.

**Characteristics analyzed**	**Women**				
	***p***	**B**	**Exp (B)**	**95% (CI)**	***p***
Age	0.777				
Smoking	0.654				
Drinking	0.497				
Hypertension	0.228				
Brain infarction	0.719				
History of antithrombotic	0.419				
Head trauma event	0.753				
Length of symptoms disappeared or alleviated	0.809				
Duration of drainage catheter	0.783				
Preoperative volume	0.277				
Interval from onset to admission	0.537				
Reduction of hematoma cavity	0.742				
Diabetes	0.985				
Use of urokinase	0.508				
Unilateral/bilateral hematoma	0.572				
AC/VP shunt	0.253				
Headache	0.663				
BMI	0.698				
Primary onset	0.421				
Frequency of urokinase used	0.665				
Unilateral/bilateral operation	0.855				
Postoperative volume	0.189				
Preoperative dysfunction of coagulation	0.399				
Dizziness	0.649				
Limb weakness	0.309				
Dysphasia	0.251				
Disturbance of consciousness	0.559				
Cardiac diseases	0.043	2.040	7.693	1.430–41.372	0.017
Length of hospital stay	0.004	0.186	1.205	1.091–1.331	<0.001

**Table 7:** Multiple linear regression analyses of characteristics related to length of hospital stay after surgery in 41–79 years old women patients with chronic subdural hematoma.

**Characteristics analyzed**	**B**	**Beta**	***P***
Duration of drainage catheter	1.132	0.280	<0.001
Complications	5.615	0.366	<0.001

## Discussion

With the continued development of scientific technologies and the increment in the older population, CSDH resulting from traumatic head injuries inevitably occurs due to traffic accidents, the propensity for falls with older age, and the use of anticoagulation medications. Although we noted discrepancies in previous reports with regard to men-women roles in the course of CSDH, a comparative investigation of the clinical characteristics regarding CSDH vis-à-vis men and women was exceedingly rare. Hotta et al. recently summarized the clinical features of CSDH in women patients (15), and they concluded that women suffered from more disorders of consciousness at admission and that their death outcomes at discharge were more frequent than for men with CSDH. Although sex represented an independent factor of consciousness disorders at admission and was a predictor of death after discharge, these authors did not take age into account in their analysis. Wang et al. also reported dissimilar results (13). Moreover, there is disagreement on the recurrence of CSDH after Burr-hole craniotomy followed by a drainage strategy (7, 16, 17, 19, 20, 27). Such controversy was the reason we chose to enroll a large sample of 1,307 participants to further investigate the impact of sex differences on the clinical characteristics of CSDH.

First, nearly all neurosurgeons have reached a consensus that CSDH occurs predominantly in the male population, and that it displays a male prevalence in all age groups (28). From an anatomical point of view, Oh et al. reasoned that cranial size differences and asymmetry between men and women may contribute to the pathogenesis of CSDH (29) and that greater exposure to injury by men and the protective estrogenic effects in women on blood vessel capillaries would also lead to the high incidence in males (30). Second, Japanese researchers from Tokai University evaluated 490 cases of CSDH and showed that consciousness disorders in women were significantly more frequent than in men, and they identified female sex as an independent predictor of disordered consciousness at admission (15, 28). However, in contrast, neither we (initially) nor Wang et al. observed a tendency for femaleness to influence consciousness at admission (13). However, when we further analyzed the data stratified for age, our results indicated that women with CSDH reflected greater disorders of consciousness at admission than male patients in the ≤ 40-year-old group, while the proportion of women with diabetes was augmented relative to that of men at 41–79 years of age. This inconsistency may be attributable to small-sample bias, and the previous authors also did not divide patients with respect to age in their studies. Third, we focused on the recurrence rate or re-operation rate with CSDH between the sexes. Investigations in 2019 that comprised 500 CSDH patients (24) and 787 patients by Motoie et al. (17) verified that men presented with higher recurrence rates of CSDH. In contrast, Mori et al. and Nakaguchi et al. uncovered no differences in the rates of CSDH recurrence between men and women (18, 23), and these data are supported by our results. Sundstrom et al. did not determine any differences in pressures inside the hematoma between the two sexes, and hematoma types occurred equally in both men and women (31). Based on the latter studies, we inferred that sex did not correlate with postoperative recurrence rate. Finally, the therapeutic outcome at discharge occupies a key role in the course of CSDH, including postoperative complications and death. Nakaguchi et al. (23) did not establish a statistical difference in hospital mortality or MRS score between men and women, conflicting with the findings from the Hotta study that indicated that female sex was a predictor of death at discharge (15). Wang et al. also suggested that male–female sex was significantly related to mortality with CSDH (13). Our results revealed that the women demonstrated more complications, a longer length of hospital stay, and higher mortality at discharge after surgery than men in the 41–79 year-old group. This suggests that the clinical characteristics between women and men with CSDH were divergent among the various ages of the patients.

With our additional analysis, we established that both the length of hospital stay and cardiac disease impacted the occurrence of complications in women aged 41–79, while the duration of drainage catheter use and complications were identified as independent risk factors and predictors for the length of hospital stay in the same group of women. The results of Davis et al. also indicated that male sex was an independent predictor of postoperative complication risk and increased hospital stay after surgery (7). This finding contrasted with our results, which may reflect the influence of more baseline comorbidities in women or the fact that the authors did not further analyze age strata. The influence of sex on postoperative complication risk is complex and multifactorial, and it may be mediated by hormonal factors or underlying disease. We suggest that the protective actions of circulating estrogens and progestins contribute to the dichotomous phenomenon whereby estrogen preserves autoregulatory functions, affects antioxidant actions, reduces neuronal excitotoxicity, upregulates the antiapoptotic factor bcl-2, and activates the mitogen-activated protein kinase pathways in women (32). We do, however, acknowledge that CSDH is prevalent in older women who have entered the postmenopausal period and have thereby lost hormonal protection.

Although the strengths of our trial included a large sample size of 1,307 patients—providing robust statistical power that enabled us to detect a difference between the sexes—additional research is urgently required to elucidate the relationship between sex and the risk of complications after the implementation of therapeutic procedures for CSDH. We posit that a clearer relationship between sex and the clinical characteristics of CSDH will be clinically useful in identifying those patients at increased risk and in predicting postoperative outcomes.

## Conclusions

This study provided a clear overview of the comparisons of the clinical features of CSDH between men and women patients, and men's statistical predominance remained evident in all the age groups. When we did not consider patient age, we ascertained that headache was the most common symptom, and that it was higher in men than in women (this included the factors of smoking and drinking history), while head trauma was not significantly different between the two sexes. We noted that only postoperative complications exhibited a difference between men and women patients after surgery. Further analysis also identified both length of hospital stay and preoperative cardiac disease as independent risk factors in women with CSDH-induced postoperative complications. After applying age as a factor, the differences in smoking and drinking history between the two groups were only observed in those patients 41–79 (*p* < 0.001, respectively) and ≥ 80 years of age. The women patients with CSDH manifested greater disorders of consciousness at admission than did men patients in the ≤ 40-year-old group, while the proportion of women with diabetes was higher than that for men in the 41–79-year group. Otherwise, the women patients after surgery showed a greater number of complications, a longer length of hospital stay, and higher mortality at discharge than men patients 41–79 years of age. Finally, the length of hospital stay and cardiac disease impacted the onset of complications in the women patients 41–79 years of age, while the duration of drainage catheter use and complications were identified as independent risk factors for length of hospital stay in this same group. We substantiated the presence of men-women differences in the clinical characteristics of CSDH before and after surgery, some of which conflicted with previous reports, and overall, we noted a lack of discrepant postsurgical outcomes as evaluated by the MRS score. Collectively, our results support the perception that sex differences constitute an unavoidable element in all CSDH patients, but we still need to pay attention to men-women disparities in certain age groups to practice favorable management and treatment of patients with CSDH.

## Data Availability Statement

The original contributions presented in the study are included in the article/supplementary material, further inquiries can be directed to the corresponding author.

## Author Contributions

YO and WL designed the research. WF, XY, and LW collected data. YO and WF drafted the manuscript. YO takes the responsibility for the integrity of the data and the accuracy of the data analysis. All authors read and approved the final manuscript.

## Funding

This work was supported by the National Natural Science Foundation of China (81502150), the Institute of Artificial Intelligence, Hefei Comprehensive National Science Center (21KT013), the Capital's Funds for Health Improvement and Research (2020-2-2045), the Capital Health Research and Development of Special (2022-2-2047), Beijing Advanced Innovation Center for Big Data-Based Precision Medicine, Beihang University, Beijing, China National Natural Science Foundation of China (81930048), Capital Characteristic Clinical Application Project (Z181100001718196), and National Key Technology Research and Development Program of the Ministry of Science and Technology of China (2014BAI04B01, 2015BAI12B04, and 2013BAI09B03).

## Conflict of Interest

The authors declare that the research was conducted in the absence of any commercial or financial relationships that could be construed as a potential conflict of interest.

## Publisher's Note

All claims expressed in this article are solely those of the authors and do not necessarily represent those of their affiliated organizations, or those of the publisher, the editors and the reviewers. Any product that may be evaluated in this article, or claim that may be made by its manufacturer, is not guaranteed or endorsed by the publisher.
